# Controlled Release of Vascular Endothelial Growth Factor from Heparin-Functionalized Gelatin Type A and Albumin Hydrogels

**DOI:** 10.3390/gels3040035

**Published:** 2017-10-09

**Authors:** Christiane Claaßen, Lisa Sewald, Günter E. M. Tovar, Kirsten Borchers

**Affiliations:** 1 Institute of Interfacial Process Engineering and Plasma Technology IGVP, University of Stuttgart, Nobelstraße 12, 70569 Stuttgart, Germany; christiane.claassen@igvp.uni-stuttgart.de (C.C.); lisa.sewald@igvp.uni-stuttgart.de (L.S.); guenter.tovar@igvp.uni-stuttgart.de (G.E.M.T.); 2 Fraunhofer Institute for Interfacial Engineering and Biotechnology IGB, Nobelstraße 12, 70569 Stuttgart, Germany

**Keywords:** controlled release, isoelectric point, carbodiimide crosslinking, albumin, gelatin type A

## Abstract

Bio-based release systems for pro-angiogenic growth factors are of interest, to overcome insufficient vascularization and bio-integration of implants. In this study, we investigated heparin-functionalized hydrogels based on gelatin type A or albumin as storage and release systems for vascular endothelial growth factor (VEGF). The hydrogels were crosslinked using carbodiimide chemistry in presence of heparin. Heparin-functionalization of the hydrogels was monitored by critical electrolyte concentration (CEC) staining. The hydrogels were characterized in terms of swelling in buffer solution and VEGF-containing solutions, and their loading with and release of VEGF was monitored. The equilibrium degree of swelling (EDS) was lower for albumin-based gels compared to gelatin-based gels. EDS was adjustable with the used carbodiimide concentration for both biopolymers. Furthermore, VEGF-loading and release were dependent on the carbodiimide concentration and loading conditions for both biopolymers. Loading of albumin-based gels was higher compared to gelatin-based gels, and its burst release was lower. Finally, elevated cumulative VEGF release after 21 days was determined for albumin-based hydrogels compared to gelatin A-based hydrogels. We consider the characteristic net charges of the proteins and degradation of albumin during release time as reasons for the observed effects. Both heparin-functionalized biomaterial systems, chemically crosslinked gelatin type A or albumin, had tunable physicochemical properties, and can be considered for controlled delivery of the pro-angiogenic growth factor VEGF.

## 1. Introduction

Presently, formation of fibrotic encapsulation around implants, as well as insufficient oxygen and nutrient supply in tissue engineering grafts due to poor vascularization remain fundamental limitations for clinical translation [1,2]. Bioactive hydrogels are often utilized to improve the biocompatibility of implants [3,4,5]. In particular, controlled release of pro-angiogenic growth factors from hydrogels is reported to stimulate vascularization, and thereby bio-integration of implants [5,6,7]. In the past decades, hydrogel materials that mimic features of the extracellular matrix (ECM) have been extensively studied as artificial scaffolds for tissue engineering [8,9,10] and for controlled growth factor release [8,9,10]. In this context, approaches based on biopolymers from the native tissue matrix have the advantage of the often inherent biocompatibility and biodegradability of the hydrogel-forming components [11].

Gelatin is a common matrix for tissue engineering [12,13] and controlled release systems [13,14,15,16,17,18]. Gelatins are available from different animal sources via different hydrolysis processes [19,20], which influence the physicochemical properties of the hydrogels, and release properties for encapsulated active agents. Generally, an acidic hydrolysis leads to gelatin, with an isoelectric point (IEP) at approximately pH 9.0 (gelatin type A), while an alkaline hydrolysis leads to an IEP at approximately pH 5.0 (gelatin type B) [20]. The IEP of gelatin has been described to affect the loading and release behavior of different growth factors, such as basic fibroblast growth factor (bFGF) [21,22,23,24], fibroblast growth factor 2 (FGF-2) [25], or stromal cell-derived factor 1 (SDF-1) [26]. Higher loading and lower release rates of gelatin type B compared to gelatin type A were observed [21,22,23,24,25,26], and attributed to the formation of polyion complexes of gelatin type B and various growth factors, due to the opposite charging [17,27]. Therefore, most authors, so far, used gelatin type B hydrogels to store and release pro-angiogenic growth factors, including vascular endothelial growth factor (VEGF), e.g., [21,28,29,30]; nevertheless, gelatin type A could be an interesting storage and release system for growth factors as well [21,31,32].

Besides gelatin-based materials, albumin has been extensively studied as surface coating to improve bio- and hemocompatibility of implants [5,33,34], or as a particulate drug delivery system [35,36,37]. Recent approaches also used albumin hydrogels for the controlled release of e.g., small molecule drugs [38,39,40,41]. It is expected that albumin can also form polyion complexes with various growth factors, due to its IEP at pH 4.7 [40], and thus, negative charge at neutral pH. Furthermore, crosslinked albumin-heparin gels without loading of growth factors were described to enhance endothelial cell adhesion and proliferation [3]. Additionally, loading of these materials with endothelial cell growth factor (ECGF) induced angiogenesis, respectively [36].

As shown above, both gelatin and albumin have been studied as components in a great variety of material systems for delivery of bioactive molecules. Yet, to the best of our knowledge, no release profiles are available for VEGF from carbodiimide crosslinked gelatin type A-heparin hydrogels, or carbodiimide crosslinked albumin-heparin hydrogels. In this study, we prepared hydrogels out of gelatin type A and albumin with heparin by the use of 1-ethyl-3-(3-dimethylaminopropyl) carbodiimide (EDC) as crosslinking agent. We hypothesize that carbodiimide chemistry allows the stable incorporation of heparin into the hydrogels, and that the crosslinker concentration can be used to adjust the equilibrium swelling degrees (EDS). We furthermore describe the swelling kinetic and hydrolytic stability of gelatin type A-based and albumin-based hydrogels. Moreover, we hypothesize that with this knowledge, VEGF can be sufficiently loaded to the hydrogels by swelling in growth factor solution. We expect different release behaviors for VEGF, depending on the biopolymer used for hydrogel preparation, applied crosslinker concentration, and loading conditions.

## 2. Results and Discussion

### 2.1. Heparin Functionalization of Hydrogels

**Figure 1:**
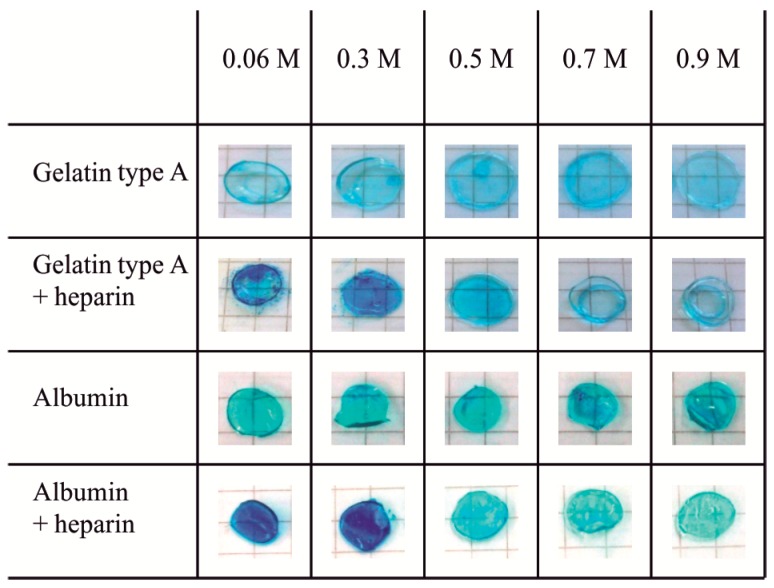
Critical electrolyte concentration (CEC) staining of gelatin type A (10 wt %) and albumin (10 wt %) hydrogels without heparin or with heparin (1 wt %) that were crosslinked with 1-ethyl-3-(3-dimethylaminopropyl) carbodiimide (EDC) (0.125 M). Hydrogels were washed for at least 5 h in PBS^+^ before staining. Alcian blue solutions (0.05%, pH 5.8) with 0.06 to 0.9 M MgCl_2_ were applied for staining. Hydrogels without heparin showed no dependence of staining on electrolyte concentration, indicating that the CEC for albumin and gelatin hydrogels was lower than 0.06 M MgCl_2_. For heparin-functionalized hydrogels, the CEC was 0.5 M MgCl_2_.

Stable incorporation of heparin into gelatin type A hydrogels and albumin hydrogels was proven by critical electrolyte concentration (CEC) staining using Alcian blue. Heparin-functionalized gels showed equal staining intensities before washing and after washing (data not shown). Figure 1 shows hydrogels of gelatin type A (10 wt %) and albumin (10 wt %) without heparin, and hydrogels containing heparin (10 wt % biopolymer + 1 wt % heparin), which were washed before staining. For both gelatin type A and albumin, the heparin-functionalized hydrogels showed an increased staining at MgCl_2_ concentrations of 0.06 and 0.3 M, compared to hydrogels without heparin. Above 0.5 M MgCl_2_, no difference in staining intensity was observed.

The positively charged dye Alcian blue stains negatively charged functional groups e.g., deprotonated carboxyl, sulfate, and phosphate groups in biopolymers, dependent on the electrolyte concentration of the solution [42]. The maximum electrolyte concentration at which staining still occurs is named the critical electrolyte concentration (CEC). The CEC is different for each type of anionic group. It expresses different affinities of the inorganic magnesium cations and the Alcian blue cations to the different anionic groups of the biopolymers. It is therefore used for differential staining of biopolymers that contain different types of anionic groups. For the CEC staining, different amounts of MgCl_2_ were added to Alcian blue solutions [42]. It was reported that carboxyl groups are stained at MgCl_2_ concentrations up to 0.1 M in vitro, while sulfate groups can be stained up to 0.8 M MgCl_2_ in vitro. The difference in the CEC of heparin-containing materials and protein-containing materials is attributed to the sulfate groups that are present in heparin besides carboxyl groups, while proteins only have carboxyl groups [42]. In the study described here, we found no influence of electrolyte concentration for albumin and gelatin hydrogels. Thus, the CEC for our protein-based hydrogel materials was below 0.06 M MgCl_2_. Heparin-functionalized hydrogels displayed a CEC of approximately 0.5 M MgCl_2_, with albumin-based hydrogels being stained stronger than gelatin type A-based hydrogels, probably due to the higher amount of carboxyl groups present per gram [20,43]. This is in good correlation to the literature CEC value for heparin, and leads to the conclusion that heparin was successfully incorporated into the hydrogel network. Alcian blue staining has also been applied to, e.g., heparin-functionalized gelatin microspheres [44], heparin-conjugated gelatin hydrogels [31], and heparinized collagen scaffolds [45], before. Adhirajan et al. and Wissink et al. used Alcian blue to localize the immobilized heparin in their microspheres or collagen scaffolds, respectively [44,45]. Both found a blue staining of heparin-functionalized materials, and no staining of the respective non-functionalized material without the addition of MgCl_2_ to the staining solution, however, they did not determine a CEC [44,45]. 

### 2.2. Physicochemical Characterization of Biopolymer Hydrogels

As described above, heparin was stably incorporated into the hydrogel network. To further characterize the hydrogels concerning their physicochemical properties, gel yield, equilibrium degree of swelling (EDS), rheological properties, and hydrolytic stability are important parameters. The gel yield is the percentage of crosslinked polymer mass divided by the total polymer mass used for preparation of the hydrogel, and gives information about the crosslinking effectiveness of the hydrogels [46]. Low gel yields can be a sign of insufficient crosslinking processes. The EDS, on the other hand, is defined as the water uptake of a gel in relation to its dry weight. It is dependent on the crosslinking density of chemically crosslinked hydrogels, and the interaction between polymer and solvent used for swelling [46]. EDS of hydrogels can therefore be seen as figure for the network density. The rheological properties, which are also a figure for the network density, are shown in the supporting information (Figure S1).

#### 2.2.1. Gel Yield

**Figure 2:**
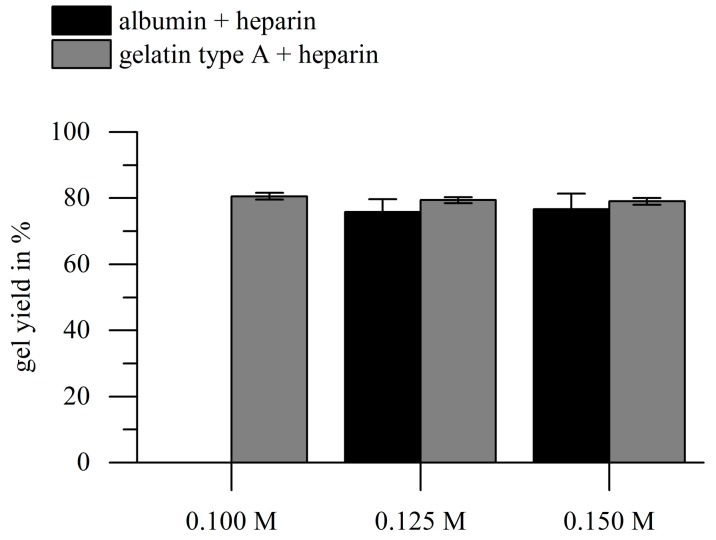
Results of the gravimetric determination of the gel yield of hydrogels. Hydrogels were prepared with gelatin type A or albumin (10 wt %), heparin (1 wt %), and different EDC concentrations. At 0.1 M EDC, no crosslinking was achieved for albumin-based gels. There was no significant influence of EDC concentration or biopolymer type on the gel yield (*p* > 0.05; *n* = 3).

To ensure sufficient crosslinking of hydrogels at different crosslinker concentrations, we calculated the gel yields for all hydrogels used for further experiments. We achieved stable hydrogel formation with the range of EDC concentrations used (0.1–0.15 M), except for albumin-heparin solutions (11 wt %), which could not be crosslinked with an EDC concentration of 0.1 M. Interestingly, the gel yields of all heparin-functionalized gels investigated in this study were approximately 80% (Figure 2). This effect can possibly be explained by the way of gel yield determination: the crosslinker mass is included in the mass of the dried hydrogels directly after crosslinking, but not in the mass of the hydrogels after washing; higher crosslinker contents cause putative lower gel yields. The weight fraction of EDC on the total dry weight in the hydrogel precursor solution was between 14.8 wt % and 20.7 wt %, depending on the used EDC concentration. Therefore, the achieved 80% is close to the maximum gel yield that is achievable. EDC concentration itself had no significant influence on the gel yield, which leads to the conclusion that if hydrogel formation was observed, crosslinking was sufficient.

To the best of our knowledge, there is no literature reporting gel yields of gelatin-based or albumin-based hydrogels via carbodiimide crosslinking. Gel yields in this study for the EDC crosslinked hydrogels were slightly smaller than the gel yields for photo-initiated radical crosslinking of methacryloyl-modified gelatin, as determined in our previous studies. There we achieved approximately 87% to 91%, dependent on the number of crosslinkable methacrylic groups available [46]. Anyhow, the weight fraction of the photo initiator Irgacure 2959 was only 0.5 wt % [46]. Therefore, the crosslinking effectiveness can be assumed to be comparable.

#### 2.2.2. Swelling Kinetics and Equilibrium Degree of Swelling

Swelling kinetics and EDS were described to be the most important parameters to control drug release of hydrogels [47]. Additionally, the swelling behavior of hydrogels in the system described here is of special interest, because the VEGF has to be loaded into the gels after the EDC-mediated crosslinking reaction has stopped, to avoid covalent coupling of VEGF into the gel. We measured the degree of swelling (DS) after 5 min, 30 min, 60 min, 3 h, 5 h, and 24 h, in order to determine the swelling kinetic for albumin-based and gelatin type A-based gels. Furthermore, we compared the EDS of albumin-based gels and gelatin type A-based gels that were prepared with different amounts of the EDC.

**Figure 3:**
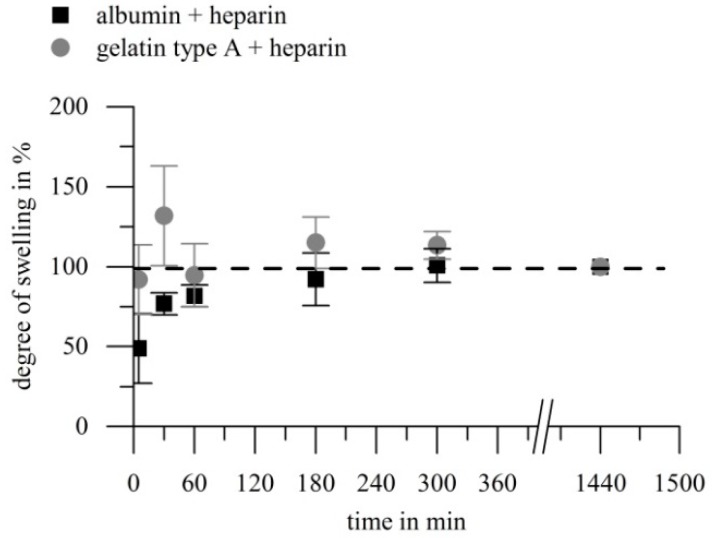
Swelling kinetics of gelatin (10 wt %) or albumin (10 wt %) hydrogels with heparin (1 wt %) crosslinked via EDC (0.125 M). The degree of swelling was determined at different time points and is denoted in % of the degree of swelling of the respective gels after 24 h. Both hydrogel types showed a fast water uptake. Gelatin-based gels passed through a hyper-swollen state after approximately 30 min, while albumin-based gels swelled continuously. Evaluation of the absolute numbers for the degrees of swelling for each gel revealed that after 60 min, no significant variation of degree of swelling (DS) was noticed. (*p* > 0.05, *n* = 4).

Figure 3 shows the comparison of swelling kinetics of albumin-heparin gels and gelatin-heparin gels, denoted as percentage of the respective DS after 24 h. Both investigated hydrogel types swelled fast, and by comparison of the absolute numbers of the DS (relating to the dry weight of the gels), no significant change was detected after 60 min for both biopolymer types. During the first 60 min, gelatin type A-heparin gels passed through a state of elevated hydration and subsequent equilibration, while the DS of albumin-heparin gels increased monotonously.

Within the literature, swelling of solvent-casted gelatin hydrogels was described to take 1 h [48], re-swelling of freeze-dried glutaraldehyde crosslinked gelatin type B hydrogels was described to take approximately 2.5 h [49], while according to [50] EDC and *N*-hydroxysuccinimide (NHS) crosslinked gelatin type A and B took 2 h to reach an equilibrium swelling state. In the last mentioned case, Kuijpers et al. prepared hydrogel-films composed of gelatin (10 wt %) which were after drying crosslinked in a solution with 0.003–0.06 M EDC and a ratio of NHS to EDC of 0.2, however they showed no re-swelling curves [50]. We found complete swelling of both, gelatin-heparin and albumin-heparin, gels within 60 min, which is in good accordance to literature for pure gelatin gels. Therefore, we propose that loading of growth factors by swelling in growth factor solution in our system should be completed after 60 min.

**Figure 4:**
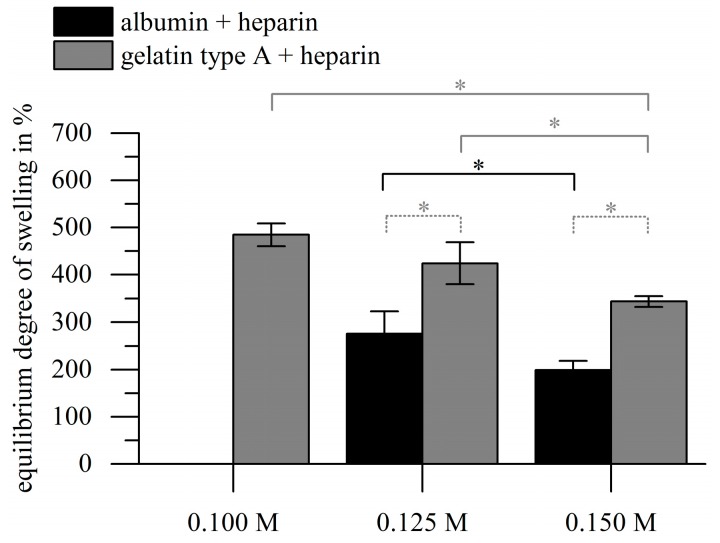
Results of the gravimetric characterization of the hydrogels. Hydrogels were prepared with gelatin type A or albumin (10 wt %), heparin (1 wt %), and different EDC concentrations. At 0.1 M EDC, no crosslinking was achieved for albumin-based gels. The EDS decreased with increasing EDC concentration for both biopolymers (asterisks with solid lines: *p* < 0.05; grey lines refer to gelatin, black line refers to albumin; *n* = 3), while the EDS of gelatin gels was significantly higher compared to the albumin gels in all cases (asterisk with dotted lines: *p* < 0.01; *n* = 3).

Beyond the swelling kinetics we also investigated the dependency of the EDS on the used crosslinker concentration and biopolymer type. The EDSs (DS after 24 h) for gelatin-heparin gels were significantly higher than for albumin-heparin gels for all crosslinker concentrations applied as depicted in Figure 4. The EDS decreased significantly in correlation with increasing EDC concentration for both materials, namely 484.7% ± 24.2% (0.1 M), 424.2% ± 44.5% (0.125 M) and 343.3% ± 11.3% (0.15 M) for gelatin-based gels, and 275.6% ± 47.0% (0.125 M) and 198.9% ± 19.7% (0.15 M) for albumin gels. No stable albumin-heparin gels were achieved with 0.1 M EDC.

In our study, the EDS was adjustable by the concentration of EDC used for crosslinking. Other studies found decreasing EDS of crosslinked gelatin hydrogels with increasing crosslinker concentration, when EDC/NHS were used [50,51]. Hydrogel films composed of crosslinked gelatin (8 wt %) had an EDS of 170–450% relating to a concentration of 0.02–0.32 M EDC and a ratio of NHS to EDC of 0.2 [52]. Other authors found that the EDS of gelatin type A-poly l lysine hydrogels was adjustable with the molar ratio between EDC and NHS, but not by EDC concentration alone [25]. This indicates that the material composition, i.e., the ratio of the amino groups and carboxyl groups involved in the crosslinking reaction, has to be considered specifically for the choice of crosslinkers. For albumin to the best of our knowledge no literature values for EDC-mediated crosslinking are available.

#### 2.2.3. Hydrolytic Stability of Hydrogels

**Figure 5:**
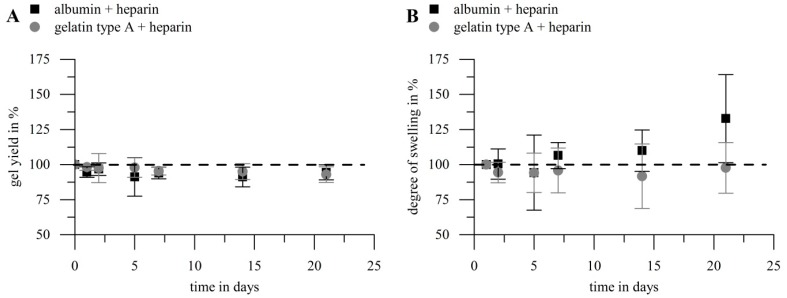
Hydrolytic stability of hydrogels prepared with gelatin (10 wt %) or albumin (10 wt %) and heparin (1 wt %) with 0.125 M EDC. Hydrogels were incubated under the same conditions as for the release of growth factor. Gel yield (**A**) and DS (**B**) were determined at different time points and are denoted as percentage of the initial values in the figure. There was no significant decrease in gel yield for both biopolymers (*p* > 0.05; *n* = 5); for the EDS a significant increase for albumin-based hydrogels was noticed (*p* < 0.05; *n* = 5), while no significant effect for gelatin type A gels was found (*p* > 0.3; *n* = 5).

The hydrolytic stability of heparin-functionalized gelatin type A and albumin hydrogels was investigated in order to gain information on potential modes of growth factor release from such gels within aqueous solution. The hydrogels were incubated under the same conditions as the samples for VEGF release. Gel yield and DS were determined after 1, 2, 5, 7, 14 and 21 days. Figure 5 shows the results, depicted as percentage of the initial gel yield or DS, respectively. The values showed no significant decrease in gel yield for both biopolymers during 21 days. The same is true for the DS of gelatin type A gels, while albumin gels showed a significant increase in DS over the investigation period.

While the hydrolytic stability of solvent casted gelatin gels [48], diisocyanate crosslinked gelatin type A [53] or gelatin type B hydrogels crosslinked with an NHS crosslinker [54] was described to be low in buffer solution at 37 °C, EDC/NHS crosslinked gelatin type A films showed high hydrolytic stability previously [52]. Solvent casted gelatin films (8 wt %) crosslinked with 0.08–0.32 M EDC and a ratio of NHS to EDC of 0.2 showed a remaining dry weight of >95% after 3 weeks [52]. This is in good agreement with our findings for EDC crosslinked gelatin-heparin hybrid hydrogels. On the contrary, EDC crosslinked albumin-heparin hydrogels were described to show a significant biopolymer loss (6.4–21.0%) in complete culture medium within 14 days at 37 °C [3]. We also observed significant hydrolysis of albumin-heparin hydrogels regarding their degree of swelling, but no decrease in gel yield. We suppose that while hydrolysis breaks down crosslinks most of the amino acid backbone of the albumin kept up the integrity of the hydrogel network. Therefore, the observed effects meet with the expectations in the sense that loss of biomolecules only appears when a high degree of degradation has been reached. This indicated that starting disintegration of the inner gel structure lead to increased swelling ability, while the amount of crosslinks was still sufficient to yield a complete network. The albumin-heparin hybrid hydrogels used in our studies therefore showed a higher stability compared to literature but a significant degradation anyway.

### 2.3. Loading and Release of Vascular Endothelial Growth Factor

**Figure 6:**
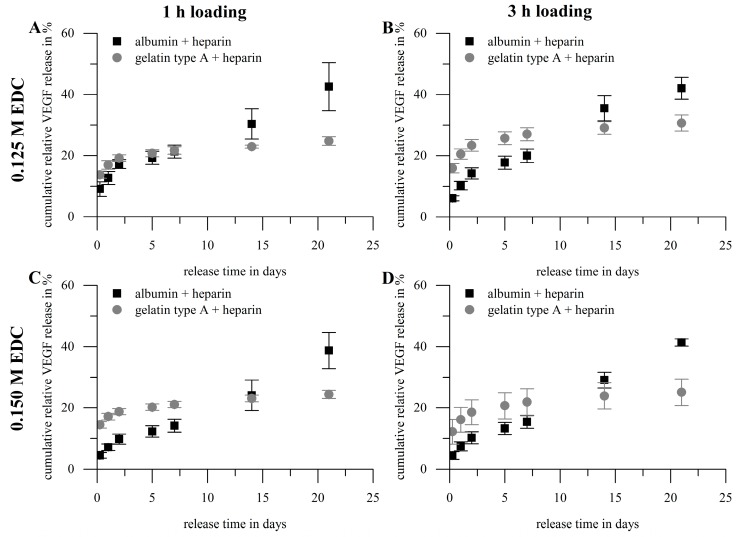
Cumulative release profiles of hydrogels prepared with gelatin type A (10 wt %) or albumin (10 wt %) with heparin (1 wt %). Loading was achieved by swelling in vascular endothelial growth factor (VEGF)-solution (1 µg VEGF per mL; loading with 0.1 µg VEGF per mg hydrogel dry weight). VEGF concentrations were normalized to the total VEGF amount used for hydrogel loading. Crosslinking and loading conditions: (**A**) 0.125 M EDC/1 h; (**B**) 0.125 M EDC/3 h; (**C**) 0.15 M EDC/1 h; (**D**) 0.15 M EDC/3 h. Gelatin type A-based hydrogels showed a higher initial release but lower overall release compared to albumin-based gels. Gelatin gels showed a diffusion controlled release curve, while release curves of albumin seemed to be diffusion controlled the first days but were afterwards similar to a zero-order release (*n* = 3).

**Figure 7:**
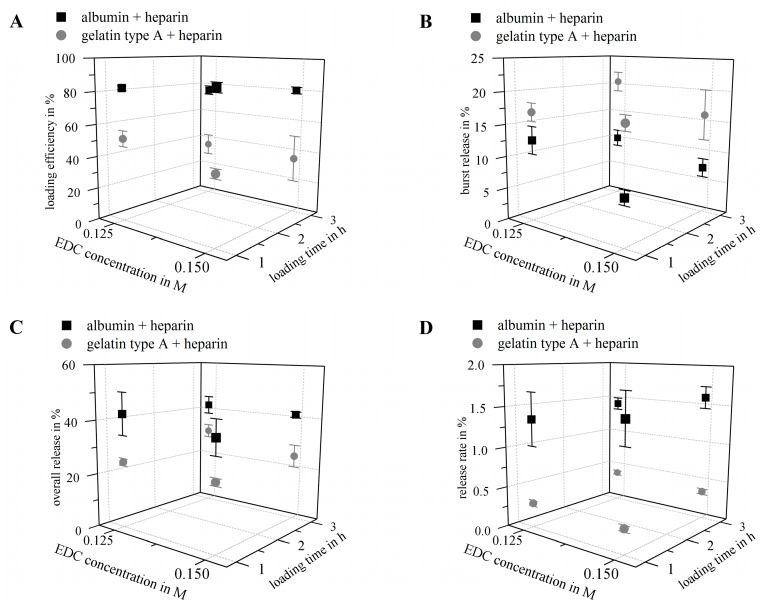
Results of key parameters for the release properties. Hydrogels were prepared with gelatin type A (10 wt %) or albumin (10 wt %) and heparin (1 wt %). For each composition, two EDC concentrations (0.125 M and 0.15 M) were used and hydrogels were loaded by swelling for 1 or 3 h in VEGF solution (1 µg VEGF per mL; loading with 0.1 µg VEGF per mg hydrogel dry weight). VEGF concentrations were normalized to the total VEGF amount used for hydrogel loading (*n* = 3). (**A**) Loading efficiency: albumin hydrogels had a higher loading efficiency compared to gelatin type A hydrogels (*p* < 0.001). For both hydrogel types the loading efficiency showed a negative correlation with the loading time (*p* < 0.01); (**B**) Burst release (release within 24 h): gelatin type A hydrogels had a higher burst release compared to albumin gels (*p* < 0.001). For albumin hydrogels, crosslinker concentration showed a negative correlation with the burst release (*p* < 0.01); (**C**) Cumulative overall release after 21 days: was higher for albumin gels compared to gelatin gels (*p* < 0.001); (**D**) Average release rate between day 7 and day 21: release rates of all albumin gels were significantly higher compared to the corresponding gelatin type A-based gel (*p* < 0.001).

Certain parameters are of special interest regarding the characterization of hydrogels as storage and release system: loading/immobilization efficiency, burst release, cumulative overall release, release rate, remaining amount of agent in the release system after the experiment and recovery rate. We prepared hydrogels composed of gelatin type A (10 wt %) or albumin (10 wt %) both functionalized with heparin (1 wt %) using two different concentrations of the carbodiimide crosslinker EDC (0.125 and 0.15 M), to investigate the effect of hydrogel composition onto the above-mentioned parameters. In preliminary studies with heparin-functionalized and non-heparin-functionalized hydrogels we observed that the cumulative overall release after 21 days was higher for albumin gels compared to gelatin gels and increased slightly for both biopolymers upon heparin-functionalization (data not shown). Thus, we investigated heparin-functionalized hydrogels further. Loading with VEGF_165_ was done by swelling dried hydrogels in a solution of 1 µg VEGF_165_ per mL for one hour or three hours, respectively. The volume used was adjusted such that hydrogels were loaded with 0.1 µg VEGF per mg dry weight. In the following the cumulative release profiles (Figure 6) of these different hydrogel compositions are described and discussed firstly, after that we take a closer look on the key figures (Figure 7) loading efficiency, burst release, cumulative overall release and release rate. Generally, all calculated values were normalized to the total VEGF amount used for loading; with this normalization it is possible to relate the released amount of growth factor directly to the amount applied for loading. A table with all values can be found in the supporting information (Tables S1–S4).

Figure 6 shows the cumulative release profiles for gelatin type A- and albumin-based gels. Gelatin-based hydrogels showed a higher initial release but a smaller overall release compared to albumin. The release profile of gelatin seemed to be completely diffusion controlled. For albumin, after a diffusion controlled initial phase, a phase similar to a zero-order release followed from day 5 on. Effects like that have been described in literature frequently and were assigned to release system degradation during release time [55], which is in good correlation with the observed increase in swelling after 5 days of incubation of albumin-based hydrogels in aqueous release medium.

Figure 7 shows the loading efficiency (A), the burst release (B), the cumulative overall release (C) and the average release rate between day 7 and day 21 (D) of the albumin-based and gelatin-based hydrogels:

- The **loading efficiency** (Figure 7A) of albumin-based hydrogels was in the range of 76.0–87.2% and significantly higher compared to the loading efficiency of gelatin type A-based gels in the range of 35.6–51.6%. The loading efficiency of both hydrogel types decreased with increasing loading time, while no general significant effect for the crosslinker concentration was observed. The highest loading was achieved at 1 h and 0.15 M EDC for albumin-based gels and 1 h and 0.125 M EDC for gelatin-based materials.
- The **burst release** (release within the first 24 h; Figure 7B) was lower for albumin-based hydrogels (7.1–12.7%) compared to gelatin type A-based gels (16.1–20.5%); that correlates to 8.1–15.4% of the loaded cargo for albumin-based gels and 32.9–56.8% for gelatin-based gels, respectively. Albumin-based gels showed the lowest bust release after 1 h loading and crosslinking with 0.15 M EDC, and a negative correlation between burst release and crosslinker concentration; while gelatin-based gels showed a similar burst release at 1 h loading and 0.125 M EDC and 3 h loading and 0.15 M EDC.
- **Cumulative release** of VEGF (release within 21 days; Figure 7C) from albumin-heparin gels was in the range of 38.7–42.5%, and therefore significantly higher than for gelatin-heparin gels (24.4–30.7%). The cumulative releases correlate to 44.4–55.4% of the loaded cargo for albumin-based gels and 47.9–85.0% for gelatin-based gels, respectively. Neither duration of loading, nor concentration of crosslinker did have significant impact on the cumulated release.
- The **average release rates** between day 7 and day 21 (Figure 7D) for albumin-based hydrogels were in the range of 1.3–1.6%, and again significantly higher compared to release rates of gelatin type A-heparin gels of 0.3–0.4%, with again no significant effect of loading time or crosslinker concentration.
- The **remaining VEGF amount** in the hydrogels after 21 days of release was only determined for gelatin-based hydrogels, since gelatin can be selectively degraded via collagenase without affecting the bioactivity of VEGF. For albumin no such an enzyme was available. The remaining VEGF-content in the gelatin-based gels was in the range of 7.2–9.8% and showed again no significant influence of loading time or crosslinker concentration.

In general, it can be noticed, that loading time and crosslinker concentration mainly showed no significant general effects, except for the **loading efficiency**. The percentage of immobilized VEGF seemed to decrease at longer loading times for both hydrogel materials according to the amounts detected in the supernatants after one or three hours loading. It can be assumed to be caused by a remodeling of the hydrogel structure. Although no significant change in hydrogel mass after 60 min was detected as described above, remodeling and hydration of polymer chains is likely to take place in a longer time frame [47].

Apart from this effect it has to be noted that the loading efficiency in our study was very high compared to the fluid uptake during the swelling in growth factor solution. While only 2.0–2.8% of the loading fluid was taken up into albumin-heparin gels according to their swelling degrees (gelatin-heparin gels: 3.4–4.2%), at least 76.0% of the growth factor present in the loading solution was loaded into the gel after one hour (gelatin-heparin gels: 35.6%). Part of this effect might be attributed to the heparin present in the gels, which is known to bind to heparin-binding growth factors such as VEGF_165_. Nakamura et al. reported an immobilization efficiency of 54.2% for VEGF on heparin-gelatin type A matrices crosslinked with glutaraldehyde, while pure gelatin matrices immobilized only 28.6% of the VEGF [31].

However, the loading efficiency in albumin-based gels was generally higher compared to gelatin type A-based materials, what we correlate with the IEP of the biopolymers. The IEP of non-crosslinked gelatin type A is described to be at pH 9.0 [20], the IEP of non-crosslinked albumin at pH 4.7 [40], while the IEP of VEGF is at pH 8.6 [56]. The crosslinking of the hydrogel systems described here is based on carbodiimide-mediated activation of carboxyl functions of the heparin molecules first and subsequent addition of the protein. In this way the crosslinking reaction can be assumed to consume a greater ratio of amino groups than of carboxyl groups of the proteins and thus the respective IEP is considered to be slightly reduced. Yet, the loading results propose that electrostatic attraction between albumin and VEGF were bigger than between gelatin type A and VEGF. Thus, gelatin, just like VEGF, was still charged positively at neutral pH, while albumin was charged negatively. The loading of various growth factors to gelatin types with different IEPs has been described to be affected by this opposite charging before [21,22,23,24,25,26,57]. Interestingly, the loading of bFGF (IEP at pH 9.6) into gelatin type B hydrogels crosslinked by glutaraldehyde was described to be higher compared to gelatin type A hydrogels [21,22], while no difference in loading of VEGF was observed [21]. This suggests that additional factors e.g., the specific chemical nature of the components definitely have to be considered besides the ionic interaction. In summary, dried albumin-heparin hydrogels were more effective in loading VEGF than dried gelatin type A-heparin hydrogels, assumingly due to the complementary net charges of gels and growth factor.

With regard to release properties of the gels we found higher **burst releases** for gelatin-based materials compared to the albumin-based gels, which can again be correlated with their IEP as described above. To the best of our knowledge there is no study investigating the release of VEGF from gelatin type A hydrogels, only results on other growth factors e.g., bFGF and FGF-2 have been published so far. The burst release of bFGF from two gelatins with different IEPs crosslinked either with EDC [23] or glutaraldehyde [24] was reported to be above 90% from gelatin type A with both crosslinking methods [23,24]. Another study investigating the effect of IEP onto burst release of FGF-2 from two gelatins crosslinked with EDC/NHS reports a burst of only approximately 30% for gelatin type A. This indicates again that probably a multitude of factors, such as polymer concentration, loading procedure, and chemical nature of growth factor and hydrogel, have significant influence on storage and release. The burst from gelatin type A gels in our study, on average 17.7% (referred to the total amount applied for loading) or 43.0% (referred to the immobilized amount), is therefore comparably small.

The VEGF-burst from albumin gels in our study was on average 9.4% (11.5% of the amount immobilized). For release of lysozyme from albumin-heparin microspheres crosslinked with glutaraldehyde, similar numbers of approximately 7% for the burst release were reported [58]. In conclusion, both hydrogel materials showed comparably low burst release for VEGF and burst from albumin-heparin hydrogels was even lower, presumably again due to the electrostatic attraction between VEGF and albumin.

The **cumulated release** of VEGF in this study after 21 days was higher for albumin-based gels compared to gelatin gels. This is in contrast to reports by Tabata et al. and Layman et al., who found lower cumulated releases from gelatin type B (which has a similar IEP as albumin) compared to gelatin type A [23,24,25]. Yet, the release kinetics confirmed the results from the degradation tests, indicating that albumin-based gels—but not gelatin-based gels—degraded from day five onwards, and therefore, released higher overall amounts of VEGF than the gelatin-based gels. Considering results from others, a broad range of cumulated bFGF release for gelatin type A hydrogels was reported, ranging from 5% [59] to 100% [24] within 21 days, while we found no data for in vitro VEGF release; our releases are within that range. Finally, both biopolymers released approximately half of their cargo during 21 days. 

The **average release rates** between day 7 and day 21 for the systems described here were higher for albumin-heparin gels compared to gelatin type A-based gels. This is in good correlation with the results for the cumulated release, and can be assigned to hydrolytic degradation in albumin gels. Concerning the vessel formation due to VEGF presence, it was reported before that micro environmental concentrations played an important role in whether VEGF-induced angiogenesis was normal or pathological [60]. Ozawa et al. proposed that hence, long-term continuous delivery of VEGF amounts below a certain micro environmental level threshold is preferable to high and fast releases [60]. Therefore, it is also important to consider the long-term release rates besides burst and cumulative release for characterizing controlled release systems. Ozawa et al. implanted 5 × 10^5^ myoblast cells in 5 µL per implantation site in the posterior auricular muscle of mice, and found normal vessel formation due to VEGF expression in the myoblasts between 2.5 ng/day per implantation side, and 35 ng/day per implantation side, and pathological vessel formation above this range [60]. Albumin-heparin gels released, on average, 1.3–1.6 ng/mg dry weight per day, gelatin type A-heparin gels released 0.3–0.4 ng/mg dry weight per day (release experiments were performed with hydrogels of approximately 5.6 mg dry weight for gelatin and 6.2 mg dry weight for albumin, respectively). Assigning the therapeutic dose found by Ozawa et al., to our release systems, approximately 4 mg wet weight for albumin-heparin hydrogels would be required, or 30 mg wet weight for gelatin-heparin hydrogels, respectively. Therefore, it can be supposed that depending on the tissue and the hydrogel weight, the releases obtained in this study were in a therapeutic range to induce angiogenesis, and the release kinetics were found to be characteristics of the hydrogel composition.

## 3. Conclusions

Gelatin type A and albumin are bio-based molecules that are already used in biomaterial applications as matrices for tissue engineering, or release systems for drugs. However, both materials were, up to now, not considered for the release of the pro-angiogenic VEGF. In this study, we used EDC-mediated crosslinking of the proteins and heparin to form hydrogels, because this zero-length crosslinker was already approved for coatings of medical devices for temporary blood contact. We observed that the water uptake for the resulting hydrogel systems could be regulated via the crosslinker concentration. Although albumin-based gels generally exhibited lower equilibrium water contents compared to gelatin-based gels, they took up higher amounts of the growth factor VEGF, when dried gels were loaded by swelling in growth factor solution. We conclude, that the unlike electrical net charge of albumin and VEGF at physiological pH additionally promoted VEGF immobilization, while in gelatin type A hydrogels, mainly the heparin was involved in VEGF immobilization; nonetheless, both bio-based hydrogel systems showed high retaining bioactivity of VEGF. Both hydrogel systems are predestinated for specific delivery requirements according to their release kinetics systems: the burst effect was lower for albumin-based gels than for gelatin type A-based gels, while the overall release after 21 days and the release rates after 7 days showed higher values for albumin-based hydrogels. This effect, and the increase of the equilibrium swelling of albumin gels after 7 days of incubation in aqueous environment, suggested that hydrogel degradation occurred during release time for crosslinked albumin, in contrast to gelatin type A hydrogels. Thus, the multitude of intermolecular interactions between the biomolecules is a chance to achieve long-lasting release of sensitive growth factors in their bioactive form, and the differential long-term stability of crosslinked hydrogels enables tuning of the release profile.

## 4. Materials and Methods

### 4.1. Materials

Recombinant human albumin, bovine serum albumin (BSA), 1-ethyl-3-(3-dimethylaminopropyl)carbodiimide (EDC), Tween-20, phosphate buffered saline containing calcium and magnesium ions (PBS^+^, pH 7.4), 2-(*N-*morpholino)ethanesulfonic acid (MES) sodium salt, and magnesium chloride hexahydrate, were purchased from Sigma Aldrich (Munich, Germany). Gelatin type A (porcine skin, ~233 bloom, 2.8 mPa s) was obtained from Gelita (Eberbach, Germany). Heparin sodium salt was purchased from Celsus (Celsus Laboratories Inc., Cincinnati, OH, USA). Alcian blue 8GX was purchased from Merck Millipore (Billerica, MA, USA). Human recombinant VEGF_165_ (rhVEGF_165_) was purchased from Morphoplant (Morphoplant GmbH, Bochum, Germany), VEGF-ELISA from PeproTech (Rocky Hill, NJ, USA). Collagenase type II was purchased from Worthington (Worthington Biochemical Corporation, Lakewood, NJ, USA). Buffers required for the VEGF-ELISA were prepared with a 0.1 M phosphate buffer, without magnesium and calcium ions (PBS^−^, pH 7.2).

### 4.2. Preparation of Heparin-Functionalized Hydrogels

Hydrogels with initial biopolymer concentration of 11 wt % (1 wt % heparin and 10 wt % gelatin or albumin) were prepared using different concentrations of carbodiimide crosslinker EDC. For gelatin-based hydrogels, gelatin, heparin, and EDC were dissolved in PBS^+^ (pH 7.4) at 37 °C; for albumin hydrogels, all components were dissolved in MES-buffer (0.1 M, pH 4.5) at 37 °C. The carboxylic acid groups of heparin were activated, firstly, by mixing heparin- and EDC-solution for 5 min at room temperature (RT). Afterwards, the gelatin or albumin solution was added to the heparin-EDC solution, and transferred into a cylindrical cast (1 mm × 30 mm). The crosslinking reaction took place at 37 °C, and in a humidified atmosphere for 1 h (gelatin) or 3 h (albumin). The crosslinked hydrogels were cut into the desired shape for the following experiments, and were taken out of the cast. Hydrogels for reference without heparin were prepared alike, using 10 wt % of albumin or gelatin.

### 4.3. Investigation of Heparin Functionalization

The stable incorporation of heparin in the hydrogels was investigated in accordance to the method of CEC staining introduced by Scott and Dorling [42]. A stock solution containing 0.05 wt % Alcian blue 8GX in 0.025 M acetate buffer at pH 5.8 was prepared. By adding appropriate amounts of MgCl_2_, different staining solutions were prepared with final concentrations of 0.06, 0.3, 0.5, 0.7, and 0.9 M MgCl_2_. The hydrogels were prepared as described above, washed, and dried. The washed and dried hydrogels were incubated for at least 12 h in the CEC-staining solutions at RT, washed, and photographed.

### 4.4. Gravimetric Characterization: Gel Yield and Degree of Swelling

Gel yield and DS were evaluated gravimetrically, as described in [46], in dependence of the used EDC amount. Hydrogels were prepared as described above, and cut into four pieces. These hydrogel pieces were dried overnight at 60 °C at 200 mbar and weighed (weight (biopolymer)). Afterwards, hydrogels were washed in 3 mL PBS^+^ at 37 °C for 5 h. Meanwhile, the buffer was changed five times. Washed hydrogels were dried again, as described before, and weighed (weight (crosslinked biopolymer_dry_)). These washed and dried hydrogels were then swollen in 3 mL PBS^+^ at 37 °C and weighed (weight (crosslinked biopolymer_swollen_)). Crosslinked gelatin and albumin hydrogels were weighed after 5 min, 30 min, 1 h, 3 h, 5 h, and 24 h swelling, for evaluation of the swelling kinetics. The gel yield (in %) and the DS (in %) were calculated as follows:

(1) gel yield = weight(crosslinked biopolymer)/weight(biopolymer)×100%

(2) $$\begin{array}{l} \mathrm{DS}\;=\;[\mathrm{weight}(\mathrm{crosslinked}\;{\mathrm{biopolymer}}_{\mathrm{swollen}})\\ -\mathrm{weight}(\mathrm{crosslinked}\;{\mathrm{biopolymer}}_{\mathrm{dry}})]/\mathrm{weight}(\mathrm{crosslinked}\;{\mathrm{biopolymer}}_{\mathrm{dry}}) \end{array}$$

### 4.5. Hydrolytic Stability of Hydrogels

Gel yield and DS after 1, 2, 5, 7, 14, and 21 days incubation in release medium (70 µg mL^−1^ BSA in PBS^+^) were examined to estimate the gels’ hydrolysis potential. Hydrogels with a diameter of 8 mm and height of 1 mm were prepared, washed, and dried as described above. Dry hydrogels were weighed to obtain the dry weight prior to hydrolysis. Afterwards, gels were incubated in 2 mL release medium each. Release medium was changed at the above-mentioned time points. Per time point, one gel (*n* = 3) was taken out of the release medium, weighed in the swollen state, and afterwards, dried and weighed again. Gel yield and DS were calculated as mentioned above.

### 4.6. Loading and Release of Vascular Endothelial Growth Factor

Hydrogels with a diameter of 8 mm and height of 1 mm were prepared, washed, and dried as described above. Loading was done using PBS^+^ containing 1 µg mL^−1^ rhVEGF_165_ and 70 µg mL^−1^ BSA. The volume was chosen such that 0.1 µg rhVEGF_165_ per mg hydrogel dry weight were applied. Dried hydrogels were incubated in loading solution for 1 or 3 h, at 37 °C on a shaker. Afterwards, the supernatant was collected and frozen immediately. Loading solution was replaced by 2 mL release medium (70 µg mL^−1^ BSA in PBS^+^), and the hydrogels were incubated at 37 °C on a shaker. Release medium was changed completely after 6 h, 1 day, 2, 5, 7, 14, and 21 days, and the samples were frozen immediately. The loading efficiency was calculated using the concentration of VEGF remaining in the supernatant after loading. The amount of VEGF that remained in gelatin hydrogels after 21 days of release was investigated by degradation of the hydrogels in 2 mL of 10 U mL^−1^ collagenase type II in PBS^+^ for 2 days at 37 °C on a shaker and subsequent freezing of the solution with degraded hydrogel. VEGF-content of all samples was determined by VEGF-ELISA, which was performed as described by the manufacturer.

### 4.7. Statistical Analysis

Statistical analysis was performed using a Student’s *t*-test or ANOVA. *p* values less than 0.05 were considered statistically significant. All data are presented as mean ± standard deviation. Unless stated otherwise, the value of *n* is defined as the number of independently performed experimental iterations.

## Supplementary Materials

The following are available online at www.mdpi.com/2310-2861/3/4/35/s1, Figure S1: Mechanical stiffness of albumin-heparin and gelatin-heparin hydrogels prepared with 0.125 M EDC. Tables S1–S4: Actual values for the release of VEGF.
