# Supplementary material for: Controlled Release of Vascular Endothelial Growth Factor from Heparin-Functionalized Gelatin Type A and Albumin Hydrogels
Source: Gels. 2017 Oct 9;3(4):35. doi: 10.3390/gels3040035 (PMC6318598; doi:10.3390/gels3040035)
Supplement: Supplementary file 1 [file gels-03-00035-s001.pdf]

Supporting Information

Controlled Release of Vascular Endothelial Growth Factor from Heparin-functionalized Gelatin Type A and Albumin Hydrogels

Christiane Claaßen, Lisa Sewald, Günter E. M. Tovar and Kirsten Borchers

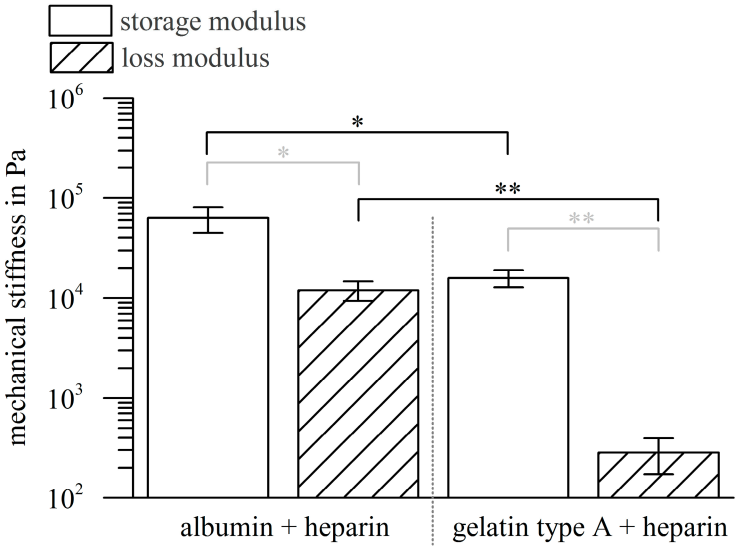

Figure S1. Results of the rheological characterization of the hydrogels. Hydrogels were prepared with gelatin type A or albumin (10 wt%), 1 wt% heparin (1 wt%) and an EDC concentration of 0.125 M. The storage modulus of both hydrogel types was significantly higher than the loss modulus; albumin-based hydrogels had higher mechanical stiffnesses compared to gelatin type A-based hydrogels. (*: $p < 0.05$; **: $p < 0.01$; n=3)

Table S1. Values for the cumulative release of VEGF from albumin-heparin hydrogels (10 wt% albumin, 1 wt% heparin). Four hydrogels were prepared with two EDC concentrations (0.125 M and 0.150 M) and hydrogels were loaded by swelling for 1 h or 3 h in VEGF solution (1 µg VEGF per mL; loading with 0.1 µg VEGF per mg hydrogel dry weight).

	**0.125 M/1 h**	**0.125 M/3 h**	**0.15 M/1 h**	**0.15 M/3 h**
Loading Efficiency	82.50%	75.97%	87.15%	80.25%
0.25 d	9.05%	6.10%	4.51%	4.46%
1 d	12.67%	10.22%	7.10%	7.40%
2 d	17.22%	14.24%	9.77%	10.24%
5 d	19.26%	17.76%	12.26%	13.24%
7 d	21.31%	19.98%	14.17%	15.41%
14 d	30.35%	35.51%	24.08%	29.07%
21 d	42.53%	42.06%	38.70%	41.37%

Table S2. Values for the cumulative release of VEGF from gelatin type A-heparin hydrogels (10 wt% gelatin type A, 1 wt% heparin). Four hydrogels were prepared with two EDC concentrations (0.125 M and 0.150 M) and hydrogels were loaded by swelling for 1 h or 3 h in VEGF solution (1 µg VEGF per mL; loading with 0.1 µg VEGF per mg hydrogel dry weight).

	**0.125 M/1 h**	**0.125 M/3 h**	**0.15 M/1 h**	**0.15 M/3 h**
Loading Efficiency	51.63%	36.12%	41.57%	35.64%
0.25 d	13.74%	15.94%	14.47%	12.18%
1 d	16.98%	20.53%	17.16%	16.10%
2 d	19.18%	23.38%	18.76%	18.53%
5 d	20.75%	25.73%	20.22%	20.68%
7 d	21.69%	27.04%	21.07%	21.93%
14 d	22.94%	29.08%	23.05%	23.88%
21 d	24.75%	30.69%	24.38%	25.07%
Collagenase Digestion	7.99%	9.80%	7.20%	7.71%

Table S3. Values for the release rates of VEGF from albumin-heparin hydrogels (10 wt% albumin, 1 wt% heparin). Four hydrogels were prepared with two EDC concentrations (0.125 M and 0.150 M) and hydrogels were loaded by swelling for 1 h or 3 h in VEGF solution (1 µg VEGF per mL; loading with 0.1 µg VEGF per mg hydrogel dry weight).

	**0.125 M/1 h**	**0.125 M/3 h**	**0.15 M/1 h**	**0.15 M/3 h**
0.25 d	36.21%	24.39%	18.05%	17.85%
1 d	4.82%	5.50%	3.45%	3.91%
2 d	4.55%	4.02%	2.68%	2.84%
5 d	1.02%	1.18%	0.83%	1.00%
7 d	1.03%	1.11%	0.96%	1.09%
14 d	1.29%	2.22%	1.42%	1.95%
21 d	1.74%	0.94%	2.09%	1.76%

Table S4. Values for the release rates of VEGF from gelatin type A-heparin hydrogels (10 wt% gelatin type A, 1 wt% heparin). Four hydrogels were prepared with two EDC concentrations (0.125 M and 0.150 M) and hydrogels were loaded by swelling for 1 h or 3 h in VEGF solution (1 µg VEGF per mL; loading with 0.1 µg VEGF per mg hydrogel dry weight).

	**0.125 M/1 h**	**0.125 M/3 h**	**0.15 M/1 h**	**0.15 M/3 h**
0.25 d	54.98%	63.75%	57.87%	48.74%
1 d	4.32%	6.12%	3.60%	5.22%
2 d	2.20%	2.84%	1.60%	2.43%
5 d	0.52%	0.78%	0.49%	0.72%
7 d	0.47%	0.66%	0.42%	0.63%
14 d	0.18%	0.29%	0.28%	0.28%
21 d	0.26%	0.23%	0.19%	0.17%
